# Dr. White’s Articles

**Published:** 1916-04

**Authors:** 


### Dr. White's Articles.

In looking about for what might be of special interest to physicians, Dr. F. S. White, of San Antonio, has been asked and has consented to write for the Texas Medical Journal a series of several articles on subjects that are not generally well enough understood by the profession. Every physician comes in contact with insanity in its early stages, but everyone does not have that intimate acquaintance with it that comes from a life-long study and practice. Dr. White is a neurologist of national reputation. He has been superintendent of both the Austin and the San Antonio asylums for the insane, and is able to give much valuable information. His first article, which will be on "Nervous Women and Gynecology," will appear in the June issue of the Texas Medical Journal, and will be followed by other articles, continuing for several months. Of course you will want to keep up with this series, but won't you be generous enough to tell other physicians about it, who may not be subscribers to this journal? The Texas Medical Journal is anxious to give extensive circulation to these helpful papers in the interest, both of the profession and of suffering mankind. You can help in doing this.

